# Dynamics in Circulating Immune Cell Subsets After Fecal Microbiota Transplantation for Recurrent *Clostridioides difficile* Infection

**DOI:** 10.14309/ctg.0000000000001008

**Published:** 2026-02-25

**Authors:** Lotte Lindgreen Eriksen, Sidsel Støy, Mette Mejlby Hansen, Emma Porsborg Gatten, Christian Erikstrup, Jens Kelsen, Benjamin H. Mullish, Julian R. Marchesi, Karen Louise Thomsen, Jens Frederik Dahlerup, Simon Mark Dahl Baunwall, Christian Lodberg Hvas

**Affiliations:** 1 Department of Hepatology and Gastroenterology, Aarhus University Hospital, Aarhus, Denmark;; 2 Department of Clinical Medicine, Aarhus University, Aarhus Denmark;; 3 Department of Clinical Immunology, Aarhus University Hospital, Denmark;; 4 Division of Digestive Diseases, Department of Metabolism, Digestion, and Reproduction, Faculty of Medicine, Imperial, London, UK;; 5 Department of Gastroenterology and Hepatology, St Mary's Hospital, Imperial College Healthcare NHS Trust, London, UK.

**Keywords:** fecal microbiota transplantation, *Clostridioides difficile*, immunology, T lymphocytes, regulatory, flow cytometry

## Abstract

**INTRODUCTION:**

Fecal microbiota transplantation (FMT) is effective for recurrent *Clostridioides difficile* infection (rCDI). Adverse reactions to FMT occur early, and cellular immune responses after FMT may contribute to effects and reactions. We compared early changes in peripheral immune cell subsets and clinical outcomes in patients with rCDI who received either FMT and antibiotics or antibiotics alone in a randomized trial.

**METHODS:**

Thirty-five patients with rCDI were randomized to vancomycin and FMT (n = 20) or vancomycin alone (n = 15). Blood samples were drawn before (wk0) and 1 week (wk1) after treatment. In 3 additional patients, blood samples were drawn before and 24 hours and wk1 after FMT. Adaptive and innate immune cell subsets and gut-homing memory (CD45RO^+^integrinβ7^+^) and effector (CD45RO^-^integrinβ7^+^) T cells were analyzed by flow cytometry.

**RESULTS:**

FMT induced subtle changes in immune cell subsets with no clear pattern from wk0 to wk1. The Treg fraction tended to decrease after FMT, and a similar decrease at 24 hours indicated rapid T regulatory cells dynamics. Natural killer T (NKT) cells increased during the first 24 hours and returned to baseline level at wk1. Regardless of FMT, patients with clinical resolution from rCDI had a decrease in nonclassical monocytes and a shift in gut-homing memory to effector cells at wk1.

**DISCUSSION:**

In rCDI, FMT induced subtle and transient dynamics in peripheral immune cell subsets. T regulatory cells and NKT cells seemed responsive and should be further studied. Cure of *Clostridioides difficile* infection may be associated with an increase in circulating gut-homing T cells.

## Graphical abstract

**Figure:**
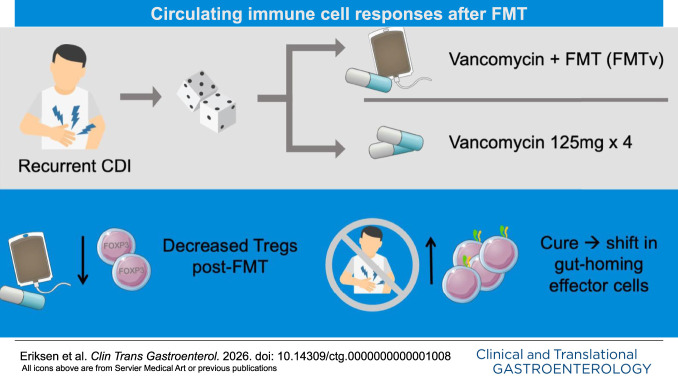

## INTRODUCTION

Fecal microbiota transplantation (FMT) is an effective treatment for both primary and recurrent *Clostridioides difficile* infection (rCDI) and superior to antibiotic treatment alone (1,2). Most patients improve promptly after FMT, and the treatment is safe and well-tolerated even in immunocompromised patients (3,4). Although some aspects of the mechanism of action behind FMT have been elucidated (5,6), the immunological impact remains poorly understood.

*C. difficile* is a Gram-positive, toxin-producing, spore-forming bacteria and a major cause of antibiotic-associated diarrhea. Antibiotics impair the intestinal microbiota allowing the *C. difficile* spores to germinate and cause disease (7). FMT results in a prompt engraftment of a donor-like microbiota in the recipient (8). Engraftment and restoration of a normal gut microbiota prevents *C. difficile* from germinating, in part through increased valerate production and restoration of bile acid metabolism (9,10).

During CDI, *C. difficile* toxins A and B are produced and cause epithelial injury and inflammation (11). An impaired antitoxin T-cell response has been reported in patients with rCDI (12). It is conceivable that an impaired toxin-induced T-cell response may be restored by FMT (13). Murine studies indicate that T regulatory cells (Tregs) are important for FMT-mediated resolution of CDI (14). Furthermore, FMT seems to restore intestinal homeostasis by reducing proinflammatory cytokines and antigen presentation in the murine intestine (15). In human, fever is a common adverse reaction to FMT (16,17), suggesting that FMT induces an immune response. FMT-induced changes in immune cell subsets have not previously been examined in a prospectively monitored patient population.

The aim of this study was to use biological samples from a randomized clinical trial to examine the impact of FMT on circulating immune cell subsets from peripheral blood in patients with rCDI and compare with the changes in patients with rCDI who received standard treatment with antibiotics. Furthermore, we evaluated whether the changes were related to treatment effect.

## METHODS

### Patients

In a randomized active-comparator, open-label clinical trial, patients with rCDI were included as previously described (1). In brief, 64 patients were included based on the following criteria: older than 18 years; 3 or more liquid stools (Bristol 6–7) per day; a positive polymerase chain reaction (PCR) test result for CD toxin A, toxin B, or binary toxin; and at least 1 prior treatment course with vancomycin or fidaxomicin. Other causes of diarrhea were ruled out. Patients were randomized to either FMT preceded by 4–10 days of vancomycin 125 mg 4 times daily (FMT precipitated by vancomycin [FMTv]), 10 days of vancomycin 125 mg 4 times daily alone (vanco), or 10 days of fidaxomicin 200 mg twice daily. For this study, we included the patients who received FMTv or vancomycin alone. To further allow investigation of short-term kinetics in the immune response, we included 3 patients with rCDI who received open label FMT in a separate observational study, independent of the randomized trial. All FMT were provided as liquid-suspension cryobag FMT, derived from 50 g of feces from healthy volunteers (18). The randomized study was approved by the Central Denmark Region Ethics Committees (j.no. 1-10-72-257-15 and 1-10-72-12-24) and registered with the Danish Medicines Agency (j.no. 2015092214), Danish Data Protection Agency (j.no. 1-16-02-15-16), and EudraCT no. 2015-003004-24. The observational study was approved by the Central Denmark Region Ethics Committees (j.no. 1-10-72-191-20).

### Samples

**Figure 1.**
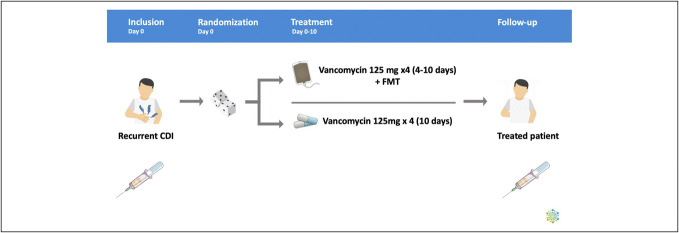
Schematic overview of blood sampling from the randomized trial. CDI, Clostridioides difficile infection; FMT, fecal microbiota transplantation.

Blood samples were collected at the day of inclusion before initiation of treatment and at wk1 after treatment termination (Figure 1). Samples from the 3 patients from the observational study were drawn at the day of and before the FMT procedure and at 24 hours and wk1. In the randomized study, peripheral blood mononuclear cells (PBMCs) were isolated from ethylenediaminetetraacetic acid whole-blood samples by Ficoll-Hypaque (GE Healthcare Biosciences, Uppsala, Sweden) gradient centrifugation, whereas sodium citrate-CPT tubes (BD Biosciences, NJ) were used in the observational study. The PBMCs were stored at −140°C until analysis.

### Multiparameter flow cytometry

At the time of analysis, PBMCs were thawed (viability >80% measured with CellDrop FL, DeNovix, WA). Cell suspensions were blocked using 0.1 mg human immunoglobulin (Privigen, CSL Behring, Lyngby, Denmark) per mL cell suspension and incubated for 15 minutes at 4°C. Afterward, surface staining was performed using optimized volumes of fluorescent-conjugated monoclonal antibodies in a Treg panel, a phenotype panel, a monocyte panel, and a homing panel (see Supplementary Table 1, Supplementary Digital Content, http://links.lww.com/CTG/B482). After 10 minutes of incubation in the dark at 4°C, cells were washed in flow running buffer and analyzed using a MACS Quant Analyzer 10 (Miltenyi Biotec, Bergisch Gladbach, Germany).

### Intracellular staining for cytokines

In 13 patients, PBMCs were thawed and incubated (2 × 10^6^ cells/mL) in culture media (Roswell Park Memorial Institute Medium + Penicillin and Streptomycin) with 10 μg/mL Golgi-blocking agent brefeldin A (BFA, Sigma-Aldrich, MO), 1 μg/mL ionomycin (Sigma-Aldrich), and 50 ng/mL phorbol 12-myristate 13-acetate (Sigma-Aldrich) for 4 hours at 37°C in a 5% CO_2_ atmosphere. Cells were harvested, and cell suspensions were blocked as described above. Afterwards, surface staining was performed using optimized volumes of fluorescent-conjugated monoclonal antibodies. After 10 minutes of incubation in the dark at 4°C, cells were washed in flow running buffer. Cell permeabilization and intracellular staining were performed as previously described (19). Cells were incubated for 20 minutes at room temperature before being washed twice to remove unbound antibodies and analyses using MACS Quant Analyzer 10 (Miltenyi). Isotype controls were included as controls.

### Gating strategy

Data files were analyzed using FlowJo version 10 (Trestar Inc., Ashland, OR). For the phenotype, Treg, homing, and Th22 panel, a lymphocyte gate and a monocyte gate were set on a forward-side scatter plot. Doublets were excluded. Alive cells were identified as Viobility^-^. CD4^+^ T cells were identified as CD3^+^CD4^+^CD8^−^, CD8^+^ T cells as CD3^+^CD4^−^CD8^+^, natural killer (NK) cells as CD3^−^CD56^+^, NKT cells as CD3^+^CD56^+^, B cells as CD19^+^, γδ T cells as γδ TCR^+^, and Tregs as CD3^+^CD4^+^CD25^+^CD127^low^FoxP3^+^. Monocytes were identified as classical CD14^+^CD16^−^, intermediate CD14^+^CD16^+^, and nonclassical CD14^−^CD16^+^. For both CD4^+^ and CD8^+^ T cell, gut-homing memory cells were identified as CD45RO^+^Integrinβ7^+^ and gut-homing effector cells as CD45RO^-^integrinβ7^+^ (see Supplementary Figures 1–3, Supplementary Digital Content, http://links.lww.com/CTG/B482). Fluorescence minus 1 for CD45RO, integrinβ7, FoxP3, and γδ TCR were used as controls. Tregs were reported as frequency of Foxp3^+^ cells of CD4^+^ T cells, while the other immune cell subsets were reported as frequency of parent.

### Statistics

Data were tested for normality using quantile-quantile-plots and histograms. A Student *t* test was used for comparison between 2 groups as data were normally distributed. The statistical analyses were performed using STATA (version 16.1, StataCorp). The results are expressed as mean and 95% CIs. A 2-tailed *P*-value < 0.05 was considered statistically significant.

## RESULTS

### Patient characteristics

From the randomized clinical trial, biological samples were available in 20 of 24 patients from the FMTv group and 15 of 16 in the vancomycin group. Clinical and biochemical characteristics of the patients are presented in Table 1. At wk1, clinical resolution with a negative *C. difficile* test was obtained in 9 (45%) patients with FMTv and 2 (13%) patients with vancomycin. The combination of clinical resolution and a negative *C. difficile* test at wk1 was used to define early global treatment effect. Further clinical outcomes have been previously described (1).

**Table 1.** Baseline characteristics

Variables	FMT and vancomycin	Vancomycin
	N = 20	N = 15
Sex (male/female)	3/17	10/5
Age (yr), median (IQR) range	68.9 [52.1–78.3] 44.9–90.6	74.1 [51.6–77.8] 21.8–92.1
Charlson Comorbidity Index score, median (range)	1 (0–5)	2 (0–7)
IBD, n (%)		
No	16 (80)	12 (80)
Yes, remission	4 (20)	3 (20)
Yes, active	0	0
Immunosuppressant therapy, n (%)	3 (15)	1 (6.7)
C-reactive protein (mg/L), median (range)	12 (1.5–147.2)	10.5 (1.8–117)
Leukocyte count (×10^9^/L), median (range)	8.9 (3.2–20.1)	7.7 (4.1–60)
Plasma albumin (g/L), median (range)	36 (24–44)	36 (19–44)

FMT, fecal microbiota transplantation; IBD, inflammatory bowel disease.

### No significant changes in immune cell subsets after FMTv

**Figure 2.**
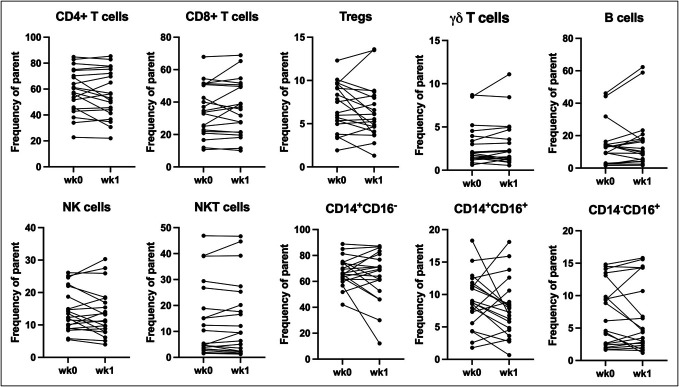
No changes in immune cell subsets in peripheral blood after FMTv. Frequencies of immune cell subsets in peripheral blood mononuclear cells measured with flow cytometry before (wk0) and after (wk1) FMTv in patients with recurrent *Clostridioides difficile* infection. Data compared using *t* test. FMTv, fecal microbiota transplantation precipitated by vancomycin; NK, natural killer.

In patients treated with FMTv most immune cell subsets did not change statistically significantly between wk0 and wk1 (Figure 2). The minor changes in immune cell subsets between wk0 and wk1 varied between the patients, and these changes were independent of patient age, sex, presence of inflammatory bowel disease, or use of immunosuppressants. Of potential importance, Tregs tended to decrease (mean ratio 0.94, 95% CI 0.79–1.07). In a subgroup of randomly selected patients, we analyzed the intracellular production capacity of IL-17A and IL-22. Only few cells expressed IL-17A, while IL-22 was undetectable (data not shown).

### Increased NK cells in patients treated with vancomycin alone

**Figure 3.**
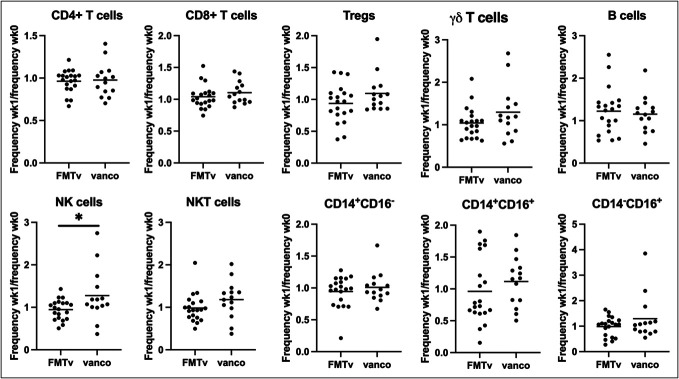
NK cells increase only in patients treated with vancomycin. Ratios of percentages between week 0 and week 1 of immune cell subsets in peripheral blood mononuclear cells from patients with recurrent *Clostridioides difficile* infection treated with FMTv or vancomycin (vanco) measured using flow cytometry. Data compared using *t* tests. Graphs shown as mean. FMTv, fecal microbiota transplantation precipitated by vancomycin; NK, natural killer.

Patients treated with vancomycin showed an increase in the frequency of NK cells at wk1 (ratio between wk1 and wk0 1.28, 95% CI 0.92–1.63) compared with patients treated with FMTv (0.95, 95% CI 0.83–1.06 in FMTv, *P* = 0.036, Figure 3). None of the other immune cell subsets showed statistically significant differences between the groups (Figure 3).

### Decreased nonclassical monocytes in patients with treatment effect

**Figure 4.**
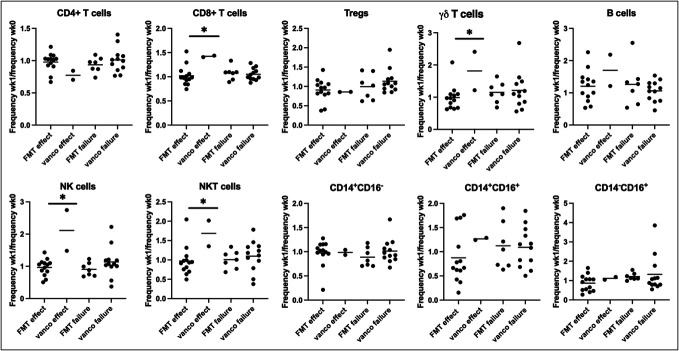
Increases in innate cell populations in patients with treatment effect after vancomycin. Changes between week 0 and week 1 in frequencies of immune cell subsets in peripheral blood mononuclear cells from patients with recurrent *Clostridioides difficile* infection treated with FMTv or vancomycin (vanco) measured using flow cytometry. Patients are divided into groups based on treatment and treatment effect. Data compared using *t* tests. Graphs shown as mean. FMTv, faecal microbiota transplantation precipitated by vancomycin; NK, natural killer.

To examine whether treatment response influenced the immune cell subsets, we compared the patients with treatment effect with those with treatment failure regardless of treatment. The frequency of CD14^−^CD16^+^ monocytes decreased in patients with treatment effect (ratio 0.80, 95% CI 0.60–1.07) compared with patients with treatment failure (ratio 1.15, 95% CI 0.92–1.42, *P* = 0.0396), but neither CD14^+^CD16^−^ nor CD14^+^CD16^+^ monocytes were significantly different between the groups. In addition, no significant differences were observed between the other cell subsets investigated. The changes in immune cell subsets were similar between patients with treatment effect and treatment failure after FMTv (Figure 4).

### Increased CD8^+^ T, NK, and NKT cells in patients with treatment effect after vancomycin

We compared early changes in patients who had effect of vancomycin alone with those who had effect of FMTv. The patients with treatment effect after vancomycin (n = 2) showed significantly increased CD8^+^ T cells (*P* = 0.018), γδ T cells (*P* = 0.0263), NK cells (*P* = 0.001), and NKT cells (*P* = 0.032) compared with patients with treatment effect after FMTv (Figure 4). No other differences were observed between the patients with treatment effect stratified by vancomycin or FMTv. In addition, no significant differences were observed between patients with treatment failure stratified by treatment.

### Cure of *C. difficile* infection is associated with changes in gut-homing CD4^+^ T cells

We examined the frequency of gut-homing memory (CD45RO^+^integrinβ7^+^) and (CD45RO^-^integrinβ7^+^) effector T cells. In both CD4^+^ and CD8^+^ T cells, we found no significant changes in the gut-homing T cell subsets comparing wk0 with wk1 after FMTv (see Supplementary Figure 4, Supplementary Digital Content, http://links.lww.com/CTG/B482). Furthermore, the gut-homing T-cell subsets did not differ between patients treated with FMTv and vancomycin (see Supplementary Figure 4, Supplementary Digital Content, http://links.lww.com/CTG/B482). However, patients with treatment effect showed a small decrease in CD4^+^CD45RO^+^integrinβ7^+^ T cells (ratio 0.97, 95% CI 0.92–1.03) compared with patients with treatment failure (ratio 1.08, 95% CI 1.01–1.16, *P* = 0.021, Figure 6). By contrast, the frequency of CD4^+^CD45RO^-^integrinβ7^+^ T cells increased in patients with treatment effect (ratio 1.099, 95% CI 0.98–1.22) compared with patients with treatment failure (0.94 95% CI 0.83–1.04, *P* = 0.039, Figure 6).

**Figure 5.**
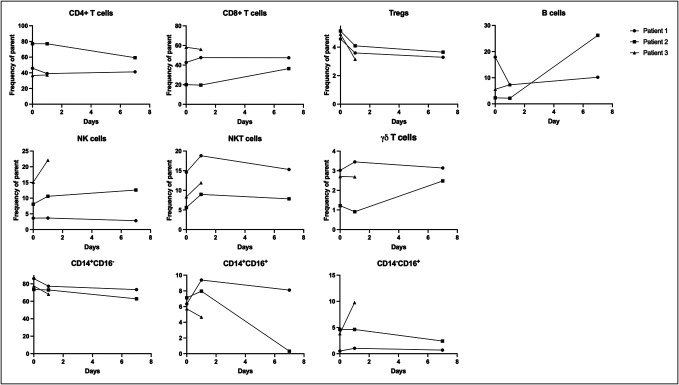
Rapid, time-dependent changes in immune cell subsets. Frequencies of immune cell subsets in peripheral blood mononuclear cells measured with flow cytometry before, 24 hours, and 1 week after FMTv in patients with recurrent *Clostridioides difficile* infection (n = 3). FMTv, faecal microbiota transplantation precipitated by vancomycin.

**Figure 6.**
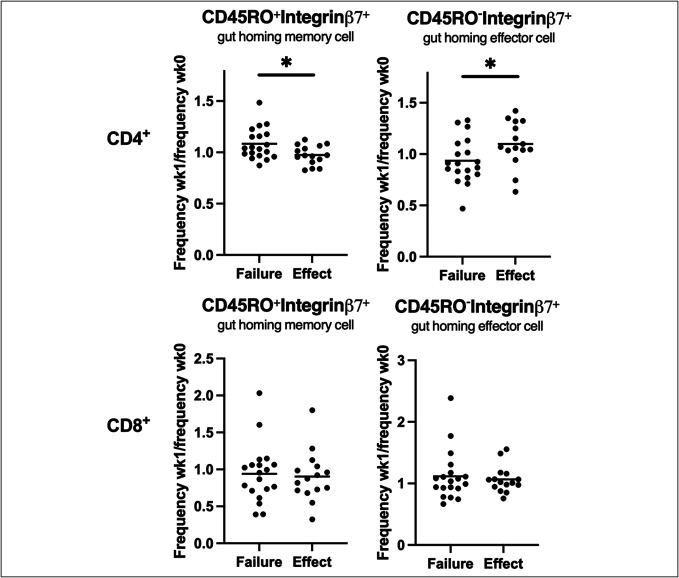
Changes in gut-homing CD4^+^ T cells associated with treatment effect. Changes between week 0 and week 1 in frequencies of CD4^+^ and CD8^+^ CD45RO^+^integrinβ7^+^ and CD45RO^-^integrinβ7^+^ T cells in peripheral blood mononuclear cells from patients with recurrent *Clostridioides difficile* infection with treatment failure and treatment effect regardless of treatment measured using flow cytometry. Data compared using *t* tests. Graphs shown as mean.

### Subgroup analysis suggests time-dependent changes

**Figure 7.**
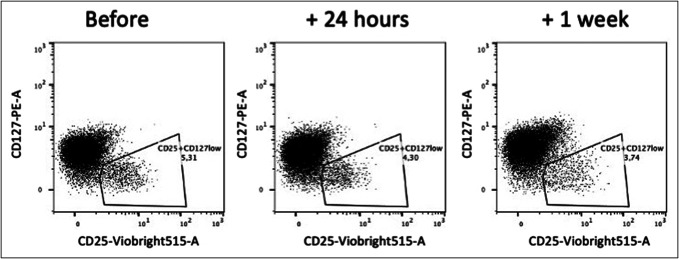
Decrease in frequencies of Tregs after FMTv. Representative flow cytometry plots of CD4^+^CD25^+^CD127^low^ cells from a patient treated with FMTv before, 24 hours, and 1 week after treatment. Data analyzed with FlowJo. FMTv, faecal microbiota transplantation precipitated by vancomycin.

In the 3 patients treated with FMTv and where 24-hour samples were obtained, we observed a decrease in circulating Tregs during the first 24 hours from the FMT (Figures 5 and 7), in line with the findings from the randomized trial. From 24 hours to wk1, the Treg frequency remained stable. Both CD4^+^ and CD8^+^ T-cell frequencies remained stable. The CD4^+^CD45RO^+^integrinβ7^+^ T cells increased slowly and continuously from baseline to wk1, whereas CD4^+^CD45RO^-^integrinβ7^+^ T cells seemed to decrease (see Supplementary Figure 5, Supplementary Digital Content, http://links.lww.com/CTG/B482). The CD8^+^ cells did not change specifically. Furthermore, we found a 5 percentage points increase in NKT cells at 24 hours, but the frequency decreased to the baseline level at wk1. No specific changes were detected in NK, B, γδ T cells, nor monocyte subsets. These exploratory data suggest that FMT may induce subtle rapid changes in circulating T-cell subsets, particularly Tregs and NKT cells.

## DISCUSSION

In this exploratory study, we investigated changes in peripheral blood immune cell subsets in patients participating in a clinical trial to compare vancomycin and FMT for rCDI. We found no significant differences in immune cell subsets between baseline and wk1, but Tregs tended to decrease after FMTv. Of interest, our illustrative subgroup of patients with blood sampling already at 24 hours after FMT (n = 3) showed a decrease in Tregs and an increase in NKT cells. This observation may be of importance, taking into consideration the swift onset of clinical effects of FMT.

To our knowledge, this is the first randomized study to describe the early effect of FMT on immune cell subsets in humans. Previous studies in patients with rCDI have focused on changes after FMT in *ex vivo* stimulated immune cells (12,13). One previous study performed a deep phenotyping of immune cell subsets, but the study only included 4 patients and no control group (20).

Overall, the immune cell subsets showed subtle changes after FMT and were not different from the changes observed in patients treated with vancomycin alone. A significant change in gut-homing CD4^+^ T cells may be important for the treatment effect. The patients with treatment effect of vancomycin (n = 2) showed an increase in CD8^+^ T, NK, NKT, and γδ T cells. Our data support the hypothesis that patients treated with vancomycin alone are dependent on a sufficient innate immune response to achieve resolution (21) unlike patients treated with FMTv.

In accordance with this study, peripheral blood immune cell subsets remained stable over time in patients with malignant melanoma treated with FMT (22). FMT induced changes in activation markers and some subpopulations (22,23), and these changes differed between treatment responders and nonresponders. These studies suggest that FMT does not affect the distribution of peripheral immune cell subsets, but instead induces changes in the activation states, i.e. the functions of the cells. Similarly, peripheral soluble inflammation markers remained stable 4 weeks after FMT in patients with rCDI, except for fibroblast growth factor-19 which increased after FMT (24).

This study was based on a well-described patient group included in a clinical trial (1). In line with other studies, the baseline frequency as well as the changes in immune cell subsets showed a large variation between the patients (12,13,25), suggesting that patients with rCDI constitute a heterogeneous group. The sex distributions were very different between the 2 groups, but neither the baseline frequencies nor the variations were associated with sex, age, presence of inflammatory bowel disease, or use of immunosuppressants.

In the randomized trial, the primary outcome was the combination of clinical resolution and a negative *C. difficile* test. Because all patients with clinical resolution and a positive *C. difficile* test had negative tests at 26 weeks without further treatment and because immune-related reactions to FMT may happen early, we focused on patients with clinical resolution at week 1 for this study.

The gut mucosal immune cells may be directly affected by FMT, although we only observed subtle changes in peripheral blood. We did not have access to colonic biopsies from the patients because the colon was found too vulnerable to perform endoscopy without high risk of complications. Costello et al (26) examined both lamina propria and peripheral blood immune cell subsets in patients with ulcerative colitis treated with FMT. They report an increase in peripheral blood CD4^+^CD45RO^+^integrinβ7^+^ T cells at week 8 after FMT treatment. In line with our study, no other changes in immune cell subsets were associated with the use of FMT. Two other studies in patients with ulcerative colitis examined colonic Tregs after FMT. One study found an increase in Tregs at week 12 (27), whereas the other found a decrease at week 4 (28). These changes could indicate that the induced changes in immune cell subsets are time-dependent. Our exploratory data from 3 patients support that changes in immune cell subsets may occur rapidly, as indicated by the marked decrease in circulating Tregs within the first 24 hours after FMT. Furthermore, a similar, but not significant, decrease was observed in both patients treated with FMTv and in patients with treatment effect in general. This decrease in Tregs may result from depletion and redistribution to the gut mucosa, but further studies are needed to investigate the role of Tregs in the orchestrated immune response to FMT.

FMTv is highly effective in treating rCDI and superior to vancomycin alone (1,29). In a randomized study, this will inevitably lead to very few patients with treatment effect after vancomycin, imposing limitations to the degree of detail with which the observed changes related to treatment effect can be interpreted. The difference in treatment effect between FMTv and vancomycin alone was even more pronounced at 8 weeks. The statistical test with treatment effect at 8 weeks was similar to testing for treatment at week 1. Unfortunately, the small sample size results in mostly insignificant findings. Because FMT is highly effective, it would be unethical to increase the sample size, but the results of this study may help guide further studies of the immune response to FMT.

In conclusion, we observed subtle changes in peripheral immune cell subsets during the first week after FMTv in patients with rCDI. Our exploratory data suggest a possible rapid response in Tregs and NKT cells after FMT, and these findings should be further pursued in larger-sized focused studies. An increase in levels of circulating innate immune cells seems associated with treatment effect in patients treated with vancomycin alone, but not FMTv. Treatment effect may be associated with a shift toward gut-homing T effector cells indicating that the effects of FMT may be restricted to the gut mucosal compartment. Further studies are needed to examine the effect of FMT on mucosal immune cells and immune cell functions.

## CONFLICTS OF INTEREST

**Guarantor of the article:** Christian Lodberg Hvas.

**Specific author contributions:** C.L.H., S.M.D.B., J.F.D., J.K., C.E., M.M.H., and E.P.G. were responsible for the clinical studies and collection of samples. L.L.E., S.S., M.M.H., K.L.T., J.F.D., S.M.D.B., and C.L.H. designed the research study. L.L.E. conducted the experiments and acquired the data. L.L.E. and S.S. analyzed the data. L.L.E., S.S., J.F.D., and C.L.H. wrote the manuscript. M.M.H., C.E., J.K., B.H.M., J.M., K.L.T., and S.M.D.B. critically reviewed the manuscript and participated with data interpretation, new ideas, and knowledge. All authors have approved the final manuscript and authorship list.

**Financial support:** Innovation Fund Denmark (j.no. 8056-00006B). C.L.H. received funding from Novo Nordisk Foundation (j.no. NNF22OC0074080). L.E. received funding from Aarhus University. B.H.M. is the recipient of a Medical Research Council Clinician Scientist Fellowship (grant number: MR/Z504002/1).

**Potential competing interests:** B.H.M. has received consultation fees from Finch Therapeutics Group and Ferring Pharmaceuticals. J.K. is a member of advisory boards in AbbVie and Janssen-Cilag, and received speakers fee from Takeda, Jansen-Cilag, and MSD. J.R.M. has received consultation fees from EnteroBiotix Ltd, Cultech Ltd, and Crescent Enterprise, Ltd.

**Study Highlights**

**WHAT IS KNOWN**

✓ FMT is an effective treatment for rCDI.
✓ The mechanisms of action behind FMT remain largely unknown.
✓ Adverse reactions to FMT often present within 24 hours.

**WHAT IS NEW HERE**

✓ In a randomized controlled trial investigating FMT for rCDI, circulating immune cell subsets are reported.
✓ T regulatory cells tend to decrease rapidly after FMT.
✓ FMT might induce subtle and rapid changes in circulating immune cell subsets.
✓ Clinical resolution is associated with a shift from memory to effector gut-homing T cells.

## Supplementary Material

## ABBREVIATIONS:

FMT: fecal microbiota transplantation
FMTv: fecal microbiota transplantation precipitated by vancomycin
NK: natural killer
PBMCs: peripheral blood mononuclear cells
rCDI: recurrent Clostridioides difficile infection
Tregs: T regulatory cells
vanco: vancomycin
