# Supplementary material for: Dynamics in Circulating Immune Cell Subsets After Fecal Microbiota Transplantation for Recurrent Clostridioides difficile Infection
Source: Clin Transl Gastroenterol. 2026 Feb 25;17(5):e01008. doi: 10.14309/ctg.0000000000001008 (PMC13193283; doi:10.14309/ctg.0000000000001008)
Supplement: Supplementary file 1 [file ct9-17-e01008-s001.docx]

**Supplementary material**

**Supplementary figure 1: Gating strategy phenotype and monocyte panel**


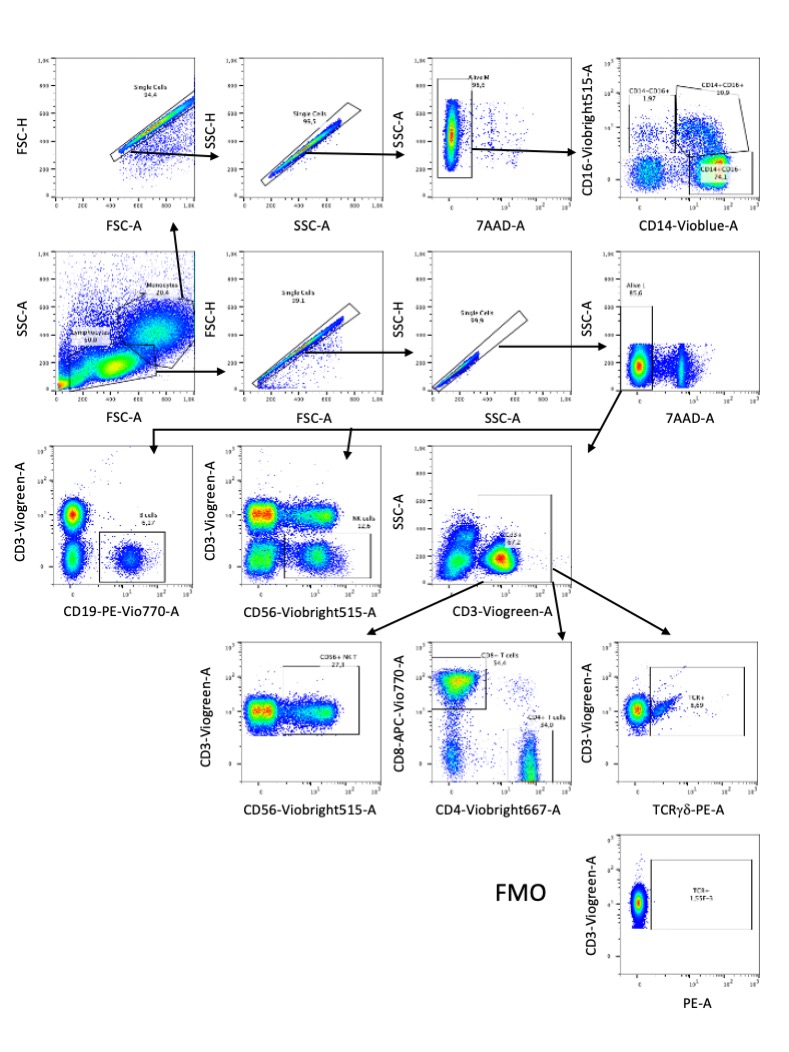


**Supplementary figure 2: Gating strategy homing panel**
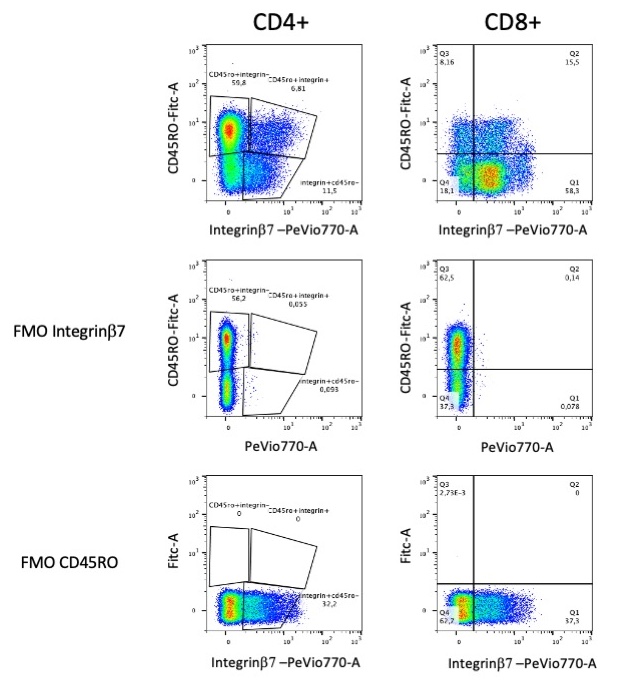


**Supplementary figure 3: Gating strategy for T regulatory cell panel**


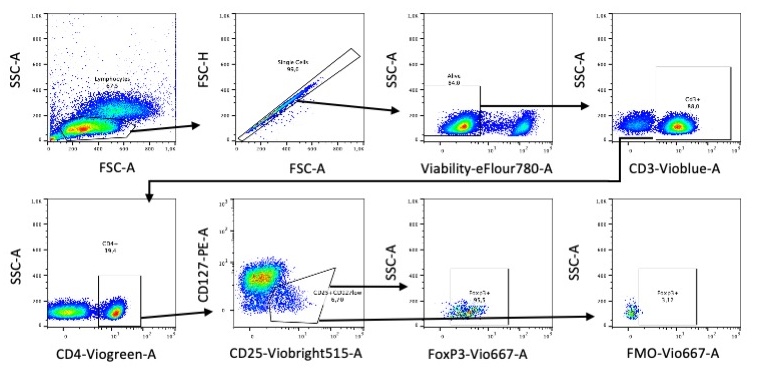


**Supplementary figure 4: No differences in gut-homing cells after faecal microbiota transplantation (FMTv)**


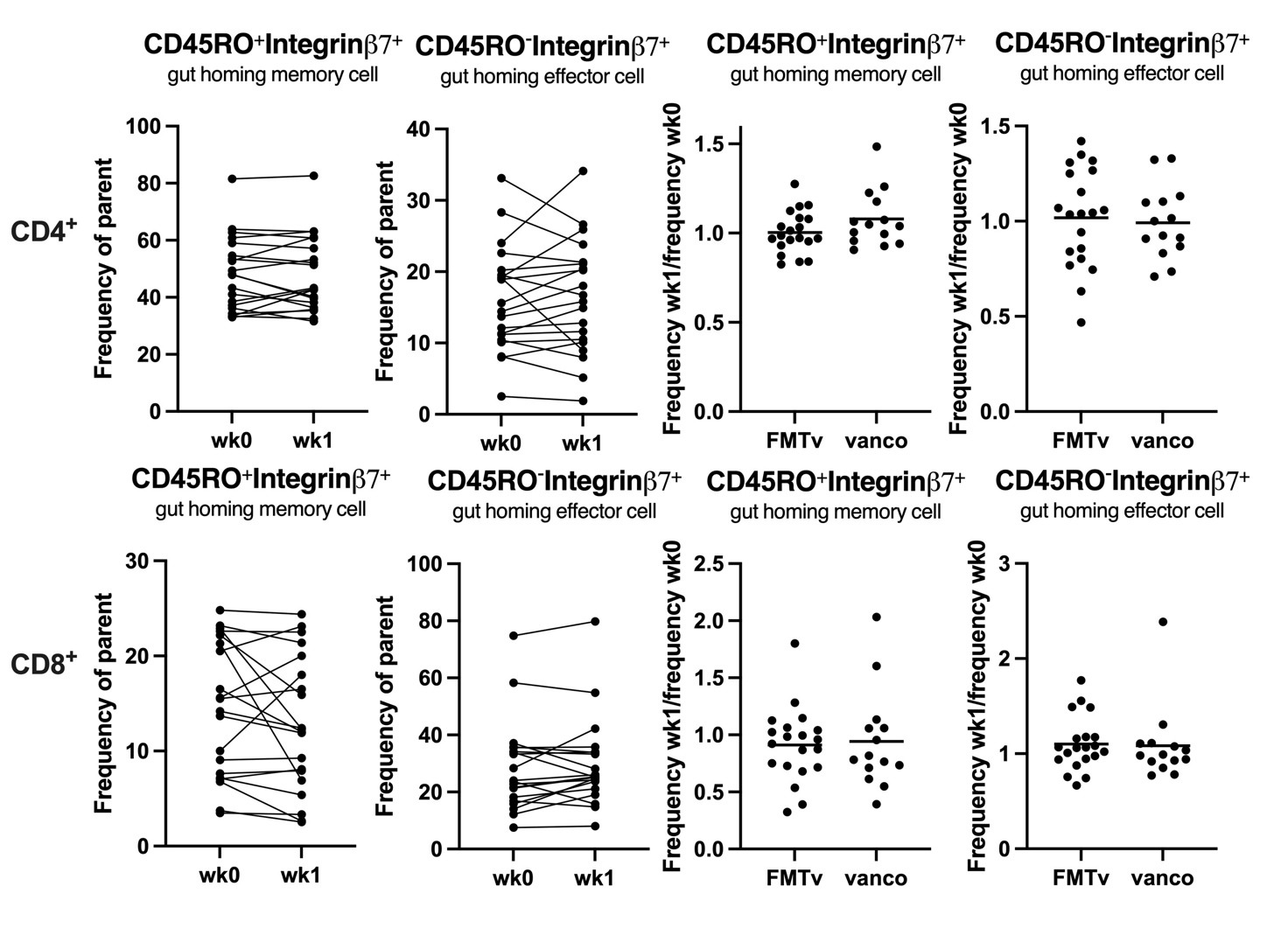


Frequencies of CD4^+^ and CD8^+^ CD45RO^+^integrinβ7^+^ and CD45RO^-^integrinβ7^+^ T cells in peripheral blood mononuclear cells from patients with recurrent *Clostridioides difficile* infection treated with FMTv measured using flow cytometry. Changes compared with patients treated with vancomycin. Data were compared using T tests. Graphs shown as mean.

**Supplementary figure 5: Kinetic changes in gut-homing T cells after faecal microbiota transplantation (FMTv)**

Frequencies of CD4^+^ (upper)and CD8^+^ (lower) CD45RO^+^integrinβ7^+^ and CD45RO^-^integrinβ7^+^ T cells in peripheral blood mononuclear cells from patients with recurrent *Clostridioides difficile* infection before (day 0), and 1 and 7 days after FMTv measured using flow cytometry. Data were compared using T tests. Graphs shown as mean.

**Supplementary table 1**

| **Antibody** | **Flourochrome** | **Manufacturer** | **Catalog number** |
| --- | --- | --- | --- |
| **Phenotype** | | | |
| CD3 | Viogreen | Miltenyi, Bergisch Gladbach, Germany | 130-113-142 |
| CCD56 | Viobright515 | Miltenyi, Bergisch Gladbach, Germany | 130-114-552 |
| TCR γδ | PE | BD Biosciences, New Jersey, United States | 555717 |
| 7-AAD |  | Miltenyi, Bergisch Gladbach, Germany | 130-111-568 |
| CD19 | PE-Vio770 | Miltenyi, Bergisch Gladbach, Germany | 130-113-647 |
| CD4 | Viobright667 | Miltenyi, Bergisch Gladbach, Germany | 130-114-532 |
| CD8 | APC-Vio770 | Miltenyi, Bergisch Gladbach, Germany | 130-110-681 |
| **Monocyte** | | | |
| CD14 | Vioblue | Miltenyi, Bergisch Gladbach, Germany | 130-110-524 |
| CD16 | Viobright515 | Miltenyi, Bergisch Gladbach, Germany | 130-119-616 |
| 7-AAD |  | Miltenyi, Bergisch Gladbach, Germany | 130-111-568 |
| **Treg** | | Miltenyi, Bergisch Gladbach, Germany | 130-122-994 |
| CD4 | Viogreen |  |  |
| CD25 | Viobright515 |  |  |
| CD127 | PE |  |  |
| FoxP3 | Vio667 |  |  |
| CD3 | Vioblue | Miltenyi, Bergisch Gladbach, Germany | 130-114-519 |
| Viability | eFlour780 | Invitrogen, Massachusetts, United States | 65-0865-18 |
| **Homing** | | | |
| CD3 | Vioblue | Miltenyi, Bergisch Gladbach, Germany | 130-114-519 |
| CD45RO | Fitc | BD Biosciences, New Jersey, United States | 555492 |
| 7-AAD |  | Miltenyi, Bergisch Gladbach, Germany | 130-111-568 |
| Integrin β7 | PeVio770 | Miltenyi, Bergisch Gladbach, Germany | 130-128-822 |
| CD4 | Viobright667 | Miltenyi, Bergisch Gladbach, Germany | 130-114-532 |
| CD8 | APC-Vio770 | Miltenyi, Bergisch Gladbach, Germany | 130-110-681 |
| **Th17** | | | |
| CD3 | Viogreen | Miltenyi, Bergisch Gladbach, Germany | 130-113-142 |
| CD45RO | Fitc | BD Biosciences, New Jersey, United States | 555492 |
| CD4 | PerCP-Vio700 | Miltenyi, Bergisch Gladbach, Germany | 130-113-228 |
| Viability | eFlour780 | Invitrogen, Massachusetts, United States | 65-0865-18 |
| IL-17A | eFlour450 | Invitrogen, Massachusetts, United States | 48-7179 |
| IL-22 | PE | R&D, Hinnerup Denmark | IC7821P |
| Isotype IgG1 | eFlour450 | Invitrogen, Massachusetts, United States | 48-4714-82 |
| Isotype IgG1 | PE | R&D, Hinnerup, Denmark | IC002P |
